# Antibacterial Effect of Thymol Loaded SBA-15 Nanorods Incorporated in PCL Electrospun Fibers

**DOI:** 10.3390/nano10040616

**Published:** 2020-03-27

**Authors:** Enrique Gámez, Hellen Elizondo-Castillo, Jorge Tascon, Sara García-Salinas, Nuria Navascues, Gracia Mendoza, Manuel Arruebo, Silvia Irusta

**Affiliations:** 1 Department of Chemical Engineering. Aragon Institute of Nanoscience (INA), University of Zaragoza, Campus Río Ebro-Edificio I+D, C/Poeta Mariano Esquillor S/N, 50018 Zaragoza, Spain; 391519@unizar.es (E.G.); helizondo_02@hotmail.com (H.E.-C.); jorgetascongarcia@hotmail.com (J.T.); 626479@unizar.es (S.G.-S.); nurian@unizar.es (N.N.); arruebom@unizar.es (M.A.); 2 Networking Research Center on Bioengineering, Biomaterials and Nanomedicine, CIBER-BBN, 28029 Madrid, Spain; gmendoza@iisaragon.es; 3 Aragon Health Research Institute (IIS Aragon), 50009 Zaragoza, Spain

**Keywords:** wound dressing, bactericidal, thymol, electrospinning, SBA-15

## Abstract

For the effective management of infected chronic wounds, the incorporation of antimicrobial drugs into wound dressings can increase their local availability at the infection site. Mesoporous silicon dioxide SBA-15 is an excellent drug carrier with tunable drug release kinetics. In this work, synthesized SBA-15 loaded with the natural antimicrobial compound thymol (THY) was incorporated into polycaprolactone (PCL) electrospun nanofibers to obtain an advanced wound dressing. Rod-shaped particles with internal parallel channels oriented along the longitudinal axis (diameter: 138 ± 30 nm, length: 563 ± 100 nm) were loaded with 70.8 wt.% of THY. Fiber mats were prepared using these particles as nanofillers within polycaprolactone (PCL) electrospun fibers. The resulting mats contained 5.6 wt.% of THY and more than half of this loading was released in the first 7 h. This release would prevent an initial bacterial colonization and also inhibit or eliminate bacterial growth as in vitro shown against *Staphylococcus aureus* ATCC 25923. Minimal inhibitory concentration (MIC: 0.07 mg/mL) and minimal bactericidal concentration (MBC: 0.11 mg/mL) of released THY were lower than the amount of free THY required, demonstrating the benefit of drug encapsulation for a more efficient bactericidal capacity due to the direct contact between mats and bacteria.

## 1. Introduction

An ideal wound dressing should protect the wound from physical damage/mechanical tear, provide adequate gaseous and nutrient exchange, and absorb the excess of wound exudates [1]. Preventing and eliminating infection is a global challenge because bacterial infection after skin injury is the main cause of failure during wound repair, increasing costs and patients’ suffering [2,3]. Local delivery of antiseptics/antibiotics is attractive as a wound infection prophylaxis because high concentrations are achieved directly at the wound bed and systemic toxicity is limited. Besides, several advantages have been reported, such as reduced side-effects, lower dosing, and maximum efficacy at the target site [4]. In addition, the local delivery of antimicrobials can decrease the possibility of emergence of bacterial resistance, since several mutations are required to acquire resistance, and a homogeneous drug concentration hinders multiple mutation selection [5]. 

For the effective management of infected chronic wounds, a promising approach is the incorporation of antimicrobial drugs into a carrier that assures its availability at the site of the infection for long periods of time without having cytotoxicity against somatic cells. Owing to favorable characteristics such as biocompatibility, large specific surface area, tunable mesoporous structure, and facile surface functionalization, mesoporous silicon dioxide has been recognized as an excellent drug carrier having tunable drug release kinetics depending on the structure, pore size, pore functionalization, and particle size [6]. In fact, mesoporous materials such as MCM-41, SBA-15 and MOF have been extensively used as drug carriers because of their exceptional and tailored sorption properties and sustained drug release ability [7]. Hu et al. [8] found that SBA-16-based 3D continuous and interconnected porous particles can reduce the diffusion resistance and facilitate transport of drugs compare to 2D cylindrical channel materials such as MCM-41. However, SBA-15 had shown higher drug loading ability than SBA-16 [9]. Two-dimensional hexagonal structured SBA-15, one of the most important types of mesoporous silicas, has large pore sizes (5–30 nm), an ordered structure, and surface silanol groups that can facilitate intermolecular hydrogen bonding with different drugs promoting their bioavailability, which is especially important for highly hydrophobic drugs; in addition, pore-mouth functionalization can avoid a premature drug release by means of molecular gates [10]. 

Due to the non-biodegradable character of SBA-15, its intravenous and intraperitoneal administration is not feasible [11] and therefore, most of its applications are focused on enteral [12], topical [13], and mucosa [14] drug delivery approaches. For instance, mesoporous materials have been previously used as carriers of poorly water-soluble molecules such as itraconazole and ibuprofen, but both drugs were released very fast from the SBA-15, when exposed to simulated gastric fluid [15]. 

*Staphylococcus aureus*, *Pseudomonas aeruginosa*, and beta-hemolytic Streptococci are the main pathogens present in both acute and chronic wounds [16]. The wound bed microbioma during infection is a complex environment, where not only multiple bacteria are present but also fungal communities can coexist [17]. The selection of the antimicrobial drug to be loaded in the wound dressing materials plays a crucial role as it is expected to combat the pathogens associated with the infected wound, considering that bacterial proliferation is a changing dynamic process and biofilm-forming pathogens are commonly associated to those non-healing infected wounds. In addition, the identification of the strain responsible for the pathogenicity is not an easy task and, therefore, a combination of culture and different molecular methods should be used to obtain a complete picture of the bacterial diversity present. Finally, the presence of antibiotic resistant strains colonizing chronic infected wounds is common [18] and that should be taken into consideration when selecting antimicrobial drugs. Due to their multiple mechanisms of action, essential oils outstand as efficient antibacterial materials having a reduced probability to induce resistances. In addition, essential oils from different plants (clove, cinnamon, rosemary, and chamomile), rich in monoterpenes and terpenoids, have shown antibacterial action against different susceptible and resistant strains, including methicillin-resistant *S. aureus* (MRSA) [19], multidrug-resistant clinical isolates of the *Burkholderia cepacia* complex [18], multi-drug resistant *Acinetobacter baumannii* [20], etc. *Origanum glandulosum* essential oil, characterized by high contents of carvacrol (CRV) and thymol (THY), was found to be strongly active against MRSA, with a minimal bactericidal concentration (MBC) of 4.7 μL/mL [21]. Besides, CRV, citral, and (+)-limonene essential oil compounds (EOCs), were found to be inhibitors of biofilm formation in multi-drug resistant strains, such as the CA-MRSA strain SC-01, whose biofilms can develop in the presence of many conventional antibiotics [22]. Not only free monoterpenes and terpenoids have shown antimicrobial action but also their encapsulation in different host matrices has provided the final construct with sustained release ability while inhibiting THY volatility. In this way, THY loaded in SBA-15 nanoparticle channels [23] have shown an antibacterial effect against *Escherichia coli* and *S. aureus*. Antifungal [24] and anti-arachnid applications have also been reported for THY immobilized within mesoporous matrices [25]. 

Electrospun nanofibers are ideal materials for topical drug delivery because long drug retention times at the wound site are achieved due to their high surface-to-volume ratio, in addition to their porous, thin, and flexile structure, which allows adequate gas exchange, avoiding wound maceration [26]. Therefore, nanofibers produced by electrospinning can provide mechanical protection, while at the same time prevent invasion of bacteria, allow gas exchange, as well as an appropriate moist wound environment prone for regeneration [27]. These properties lead to an enhancement in the rate of cell respiration, skin regeneration, moisture regeneration, removal of exudates, and haemostasis.

Polycaprolactone (PCL) is a semi-crystalline synthetic polyester that has been widely electrospun to be applied in different biomedical scenarios due to its good biocompatibility and biodegradability [28]. PCL electrospun fibres offer excellent characteristics such as high porosity and large surface area; besides, nanofibrous membranes exhibit tunable mechanical properties such as flexibility and elasticity that facilitate their application on the wound bed [29]. 

We have previously found that when comparing CRV, THY, cinnamaldehyde, eugenol, β-caryophyllene, and rosmarinic acid, CRV and THY were found to be the most active essential oil components against *S. aureus* and *E. coli* [30]. CAR- and THY-loaded PCL electrospun fibers have also shown to be able to completely eradicate bacterial pathogens, being a key issue in the antimicrobial effect observed the interaction between the PCL-based mat and bacteria [31]. We have also found that THY-loaded PCL fibers were also able to significantly reduce inflammation in an *in vitro* model [32]. Beside, Tarik et al. [33] demonstrated that drug-encapsulated mesoporous silica incorporated into nanofibrous matrices resulted in high encapsulation efficiency and controllable release profiles. In this scenario, the aim of this work is the incorporation of THY in SBA-15 nanoparticles to be loaded in PCL electrospun nanofibers as a potential bactericidal wound dressing material, showing controlled release of the adsorbed THY. 

## 2. Materials and Methods 

### 2.1. Materials

Polycaprolactone (PCL, Mn = 80,000 Da), tetraethyl orthosilicate (TEOS,≥ 99.0%), naproxen sodium salt (98–102%), phosphate-buffered saline (PBS), Tween 80, heptane (≥ 99%), Pluronic® P-123 (Mn ≈ 5800 Da), and hydrochloric acid (HCl, 37%) were purchased from Sigma-Aldrich (Madrid, Spain). Thymol (THY, 99%) was purchased from Acros Organics (Geel, Belgium). Dichloromethane (DCM, > 99%) and N,N-dimethylformamide (DMF, > 99%) were purchased from Fisher Scientific (Pittsburg, PA USA). Acetonitrile (≥ 99.9%) and formic acid (98–100%) were purchased from VWR (Barcelona, Spain). Tryptone soy agar (TSA) and tryptone soy broth (TSB) were purchased from Laboratorios Conda-Pronadisa SA (Madrid, Spain) and ammonium fluoride (NH_4_F, ≥ 98.0%) was purchased from Fluka (Buchs, Switzerland). All compounds and solvents were used without any purification.

### 2.2. Methods

#### 2.2.1. SBA-15 Particles Synthesis

Rod-shaped SBA-15 was synthesized following the method proposed by Johanson et al. [34] Briefly, Pluronic® P123 was mixed with 14 mg of ammonium fluoride in HCl 1.75 M. This solution was added to 3.25 mL of TEOS dissolved in heptane and stirred. The mixture was loaded into an autoclave and heated at 100 °C during 24 h. The resulting material was thoroughly washed and calcined at 550 ºC for 5 h to remove the organic template. 

#### 2.2.2. EOCs Loading

Before EOCs loading, SBA-15 particles were dried overnight at 100 °C in order to remove adsorbed water from the pores. EOCs load was carried out by dissolving THY in ethyl acetate (322 mg/mL), adding 100 mg of SBA-15, and keeping the obtained suspension under stirring for 1 h. The mixture was then filtered, washed four times, and dried at 37 °C for 24 h. Loading efficiency (LE) was calculated with the following formula:

LE% = (experimental loading)/(theoretical loading) × 100(1)

The experimental loading was obtained from the TGA curves as a mass balance from the difference between the initial weight, which was assigned as 100% and the residual weight after THY thermal decomposition, as previously reported [35]. 

#### 2.2.3. SBA-15 Loaded PCL Fibers

PCL fibers containing THY-loaded SBA-15 particles were prepared in a 2.2.D-500 YFlow electrospinner, using a coaxial setup. PCL was dissolved in a DMF:DCM 1:1 mixture with 10 wt.% polymer concentration and this solution was loaded in a syringe connected to the inner part of a coaxial needle. Twenty mg/mL THY loaded SBA-15 particles suspended in a 5 wt.% solution of PCL in DMF:DMC 1:1 were fed in the outer part of the coaxial needle in order to concentrate the loaded THY on the external surface of the resulting fibers. The tip was placed at 18 cm from a flat collector plate. Pumps were set at 1.0 mL/h flow rate for the inner solution and 0.5 mL/h for the outer solution, respectively. A voltage of −4.29 kV was connected to the collector plate and a positive voltage of 9.94 kV was connected to the metallic needle. The obtained material was named PCL@THY/SBA-15. The LE was calculated using the same formula given in the previous section.

#### 2.2.4. Characterization Techniques

XRD patterns were obtained using an Empyrean X-ray Diffractometer (PANalytical, Henderson, NV, USA) with Cu-Kα incident radiation at 45 kV voltage and 40 mA current. Patterns were recorded in the range from 0.5° to 8° with step size of 0.01. Scanning electron microscopy images were recorded in a SEM FEI Inspect™ F50 (FEI Company, Hillsboro, OR, USA). Samples were previously covered with a gold/platinum layer. Transmission electron microscopy images were recorded in a FEI Tecnai T20 at 200 kV (FEI Company, Hillsboro, OR, USA). Particles length and nanofibers diameters were calculated from 3 independent SEM images (n = 100) using the ImageJ software (National Institutes of Health, Bethesda, MD, USA). Thermogravimetric analyses (TGA) were carried out in a TGA/SDTA851 system (Mettler Toledo, Columbus, OH, USA) under air at a heating rate of 10 °C/min. Fourier transform infrared (FTIR) spectroscopy analyses were performed with a Bruker Vertex 70 FTIR (Bruker, Billerica, MA, USA) spectrometer equipped with a DTGS detector and a Golden Gate diamond ATR accessory. Spectra were recorded by averaging 40 scans at a resolution of 4 cm^−1^ in the 4000–600 cm^−1^ range. Nitrogen adsorption isotherms were obtained with a TriStar 3000 Micromeritics (Norcross, GA, USA) at −195.8 °C with samples degassed at 200 °C (40 °C for THY loaded samples to prevent THY degradation during outgassing) for 8 h. BET surface area was measured at the relative pressure of 0.05–0.20. Pore size distribution was determined from the adsorption isotherm using the Barrett, Joyner y Halenda method (BJH). The total pore volume was calculated at P_0_/P = 0.96.

#### 2.2.5. THY Release Assays

Slide-A-Lyzer® MINI dialysis devices (Fisher Scientific, Pittsburg, PA, USA) were used to perform the THY release experiment. SBA-15 loaded particles were dispersed under sink conditions in Phosphate Buffered Saline (PBS) containing 2 wt.% Tween solution loaded into the dialyzer. The system was stirred at 200 rpm and kept at 37 °C. The THY released was quantified by regularly sampling the buffer up to 400 h. Each time, the dialysis media were replenished with fresh solution. Release from PCL@SBA-THY nanofibers was carried out in the same way as that used for the loaded particles. THY concentration was measured by using an Ultra Performance Liquid Chromatograph (UPLC) Acquity Class (Waters Corporation, Milford, MA, USA) with a variable wavelength photodiode array detector (PDA) at 275 nm. A C-18 BEH Acquity UPLC column was employed, with a mobile phase of acetonitrile/water at 40 °C. The flow rate used was 0.3 mL/min with an injection volume of 2 µL, using naproxen as internal standard.

#### 2.2.6. Bactericidal Activity

In order to determine the antibacterial activity, THY-loaded SBA-15 particles were sterilized under UV light (λ= 254 nm, 30 minutes) and dispersed in TSB inoculated with 10^6^ CFU/mL *of S. aureus* ATCC 25293. Samples were incubated at 37 °C for 24 h and bacteria concentration after the time was determined by the standard microdilution method. Minimal inhibitory concentration (MIC) and minimal bactericidal concentration (MBC) were determined from these measurements.

For the determination of antibacterial activity of synthetized nanofibers, an adaption of ASTM E-2180 norm was employed. Fifty mL of warm TSA (47 °C) was inoculated with 50 µL of a stationary culture (10^9^ CFU/mL) of *S. aureus* ATCC 25923. Nanofiber mats were cut and sterilized under UV light (1 h each side, λ= 254 nm). Samples were placed in a 12-well plate and 1 mL of inoculated TSA (10^6^ CFU/mL) was added. Samples were incubated at 37 °C for 24 h in a closed box with sterilized water to maintain an adequate humidity. After this time, the bacteria were recovered, transferring each TSA sample to a Falcon type flask with 10 mL of TSB that was sonicated for 1 minute and vortexed for 1 additional minute. Colony forming units per milliliter (CFU/mL) were calculated from all suspensions using the standard microdilution method.

In order to study the influence of the bacteria-mats contact, enough mass to reach MBC of NFs were cut, sterilized under UV light (1 h for each side), and incubated in sterile TSB medium for 1, 3, 5, 7, 10, or 14 days. The mats were then separated from TSB and antibacterial activity was tested again. TSB with released THY collected in the supernatants (called as “exudates”) was also challenged against 10^6^ CFU/mL *S. aureus*. Both assays were carried out as previously described.

## 3. Results

### 3.1. Characterization

#### 3.1.1. Loaded SBA-15 Particles

**Figure 1:**
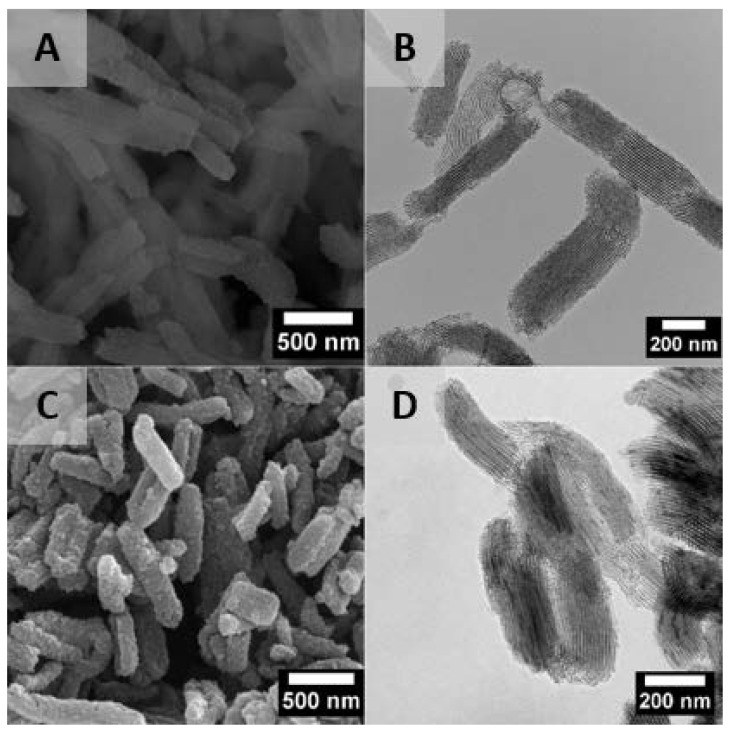
(**A**) SEM and (**B**) TEM images of pristine SBA-15 particles; (**C**) SEM and (**D**) TEM images of THY-loaded SBA particles. N = 100 particles.

The synthesized mesoporous material was rod-shaped with internal parallel channels oriented along the material axis, as can be observed in SEM and TEM images (Figure 1A,B). The mean diameter and mean length of the mesoporous rods, obtained from measuring 200 individual particles were 138 ± 30 and 563 ± 100 nm, respectively. The morphology of the mesoporous nanoparticles was preserved after drug loading, as shown in Figure 1C,D. 

**Figure 2:**
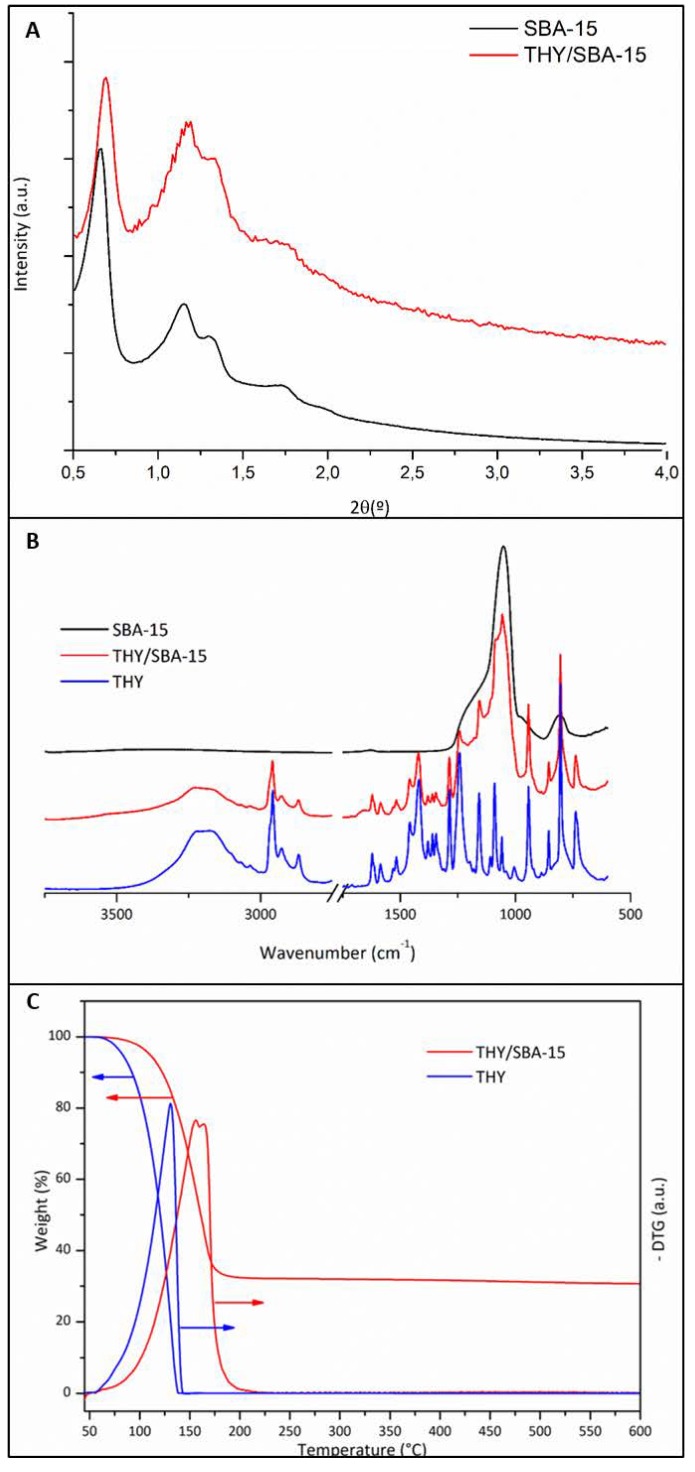
(**A**) Small Angle X-Ray diffraction pattern for SBA-15 NPs and SBA-THY NPs, (**B**) IR spectra of SBA-15 NPs, SBA-THY NPs and thymol, (**C**) TGA and DTGA of THY and THY/SBA-15 NPs.

Small angle XRD patterns presented well-resolved peaks assigned to (100), (110), and (200) diffraction planes characteristic of materials, which exhibit a structure typical of SBA-15 mesoporous molecular sieves (Figure 2A), in agreement with the previous literature [36]. The first diffraction peak is characteristic of hexagonally ordered mesopores, whereas two well-resolved low intense peaks characterize well organized mesopores in a long range. The loading of THY into the SBA-15 did not change both features mentioned. The presence of the organic compound caused a shift of the XRD diffraction peaks to higher 2 theta angles; this behaviour could be attributed to the decrease in pore size upon sample loading [37]. This result was confirmed by nitrogen adsorption. Pore sizes vary from the initial pristine material having a size distribution centred at 10.5 nm to a material with almost no pores having the remaining surface area (0.05 cm^3^/g), with a pore size distribution centred at 9 nm (Table 1) [34]. After THY loading, the specific surface was dramatically decreased, probably due to the presence of the essential oil in the mesopores and in the inter-particle spaces. 

The presence of the organic compound on the loaded samples (THY/SBA-15) was also corroborated by FTIR analysis (Figure 2B). The pristine sample spectrum shows the characteristic 1100 and 800 cm-1 bands related to asymmetric and symmetric stretching vibrations of Si-O-Si groups, respectively. The peak at 960 cm^−1^ is characteristic of the Si-OH stretching [38]. THY spectrum was included for comparison. Peaks observed between 1600 and 1400 cm^−1^ could be attributed to in-ring carbon–carbon stretching. Stretching vibration peaks for the aromatic hydroxyl groups were identified at 1250 and 1255 cm^−1^ for THY. Out of plane stretching peaks due to aromatic C–H bonds were observed in the 900–650 cm^−1^ range. Finally, the aromatic C = C stretching was identified at 800 cm^−1^ in agreement with the previous literature [39]. IR spectrum of THY-loaded particles presents, beside bands related to C-H stretching of the organic compounds in the 3000–2850 cm^−1^ region (not shown), signals in the 1700–700 cm^−1^ range superimposed to the silica bands. The THY loading in SBA-15 nanoparticles was also studied by TGA. SBA-15 is thermally stable and does not degrade under heating; only a condensation of the silanol groups occurs [40]. 

The THY- loaded particles presented a thermal degradation curve having a weight loss in the 50–220 °C range (Figure 2C) that could be attributed to the decomposition of THY located on the particles surface and inside their pores. The mesoporous silica support would increase the THY thermal stability since the pure compound start to decompose at the same temperature (around 50 °C) but it is completely decomposed at 140 °C (Figure 2C). TGA results indicated a THY load of 70.79 ± 5.21 wt.% (Table 1) that implies a loading efficiency of 80.3%. This high load would indicate that most of the essential oil component is on the SBA-15 pores and in the inter-particle spaces due to the impregnation method followed, wherein an excess of THY was used. Since those particles are incorporated into the organic electrospinning solution, a large amount of this THY excess would be extracted by the organic solvent. Therefore, to prevent a loss in the THY loading inside the SBA-15 mesopores, an excess of THY was used for the initial impregnation.

#### 3.1.2. THY/SBA-15 Loaded PCL Fibers

**Figure 3:**
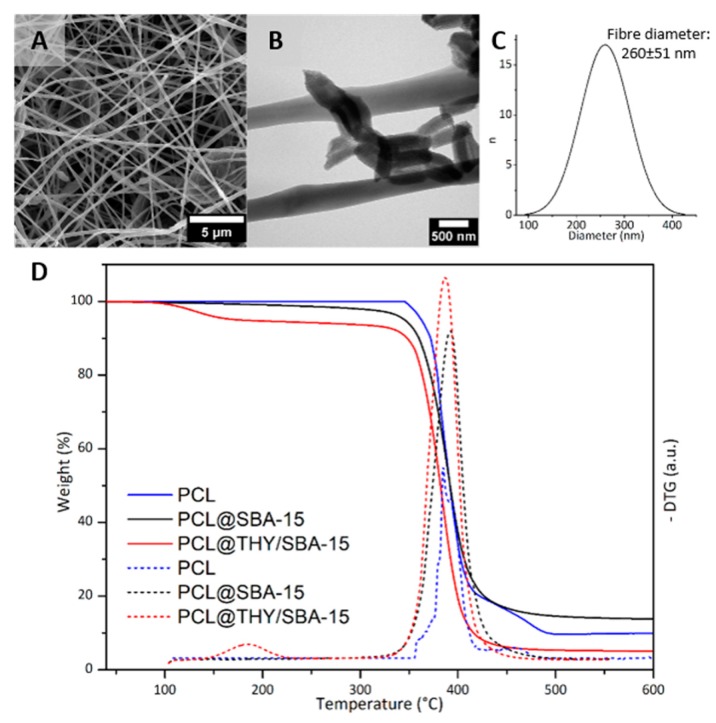
(**A**) SEM and (**B**) TEM images of PCL@THY/SBA-15 fibers; (**C**) fibers diameter histogram; (**D**) TGA curves of fibers containing loaded and un-loaded particles.

The morphology of the fibres having THY-loaded silica nanoparticles as nanofillers was studied by SEM and TEM microscopies (Figure 3A,B). The average fibre diameter of the composite mats was 260 ± 51 nm (Figure 3C), very similar to the average diameter of neat PCL fibres (262 ± 49 nm). The addition of around 5 wt.% of inorganic particles to the precursor solution did not affect fibre morphology in terms of average fibre diameter and diameter distribution. Similar results were previously obtained when introducing MCM-41 in electrospun PCL fibres with similar loadings [41]. 

TEM images (Figure 3B) confirm the presence of the SBA-15 nanoparticles into the polymeric fibres. Individual particles and some agglomerates can be observed, preferentially located close to the fibres surface. As expected, because of the relationship between fibres diameter and particle size, SBA-15 particles were mostly longitudinally oriented in the fibres. The efficacy of the use of the coaxial system to place inorganic particles as fillers preferentially on the fibre shells was previously reported [42,43].

The composition of PCL@THY/SBA-15 fibres was determined by the analysis of TG/DTG curves (Figure 3D. The decomposition process is difficult to distinguish from the thermograms, but the DTG curves showed a two-step thermal decomposition process. Considering the silica nanoparticles content in the PCL nanofibers given by TGA (4.96 wt.%) and the amount of THY in excess used to impregnate the SBA-15 nanorods, a theoretical THY loading in the final nanocomposite should be around 12 wt.%. However, as we mentioned before, a large part of the THY would be lost in the organic solvent used (DMF:DCM) during the electrospinning process. Accordingly, the final content of THY was quantified by the mass loss in the range of 70–190 °C, giving a value of 5.87 ± 0.91 wt.%, which corroborates that a large amount of THY was evaporated during the electrospinning and that the use of THY in excess is required. In the electrospinning process, volatile compounds (i.e., DMF:DCM and THY) would evaporate during fibre elongation and precipitation on the collector.

### 3.2. Thymol Release

**Figure 4:**
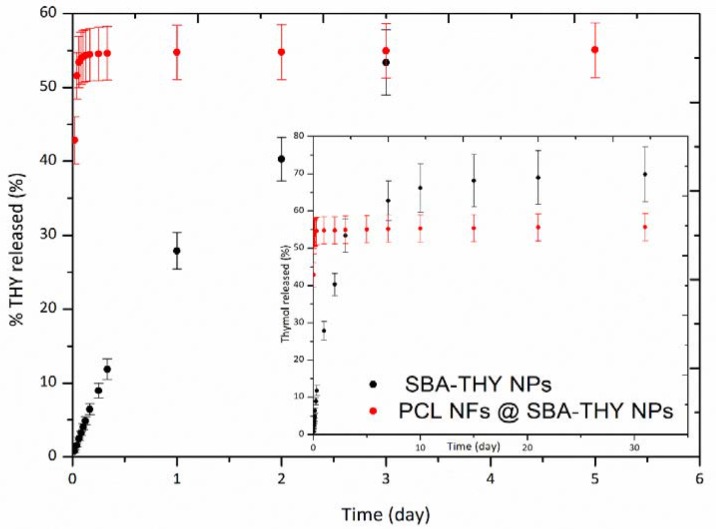
Release profile of THY form SBA-THY NPs and PCL NFs@SBA-THY NPs.

Figure 4 compares the THY release kinetics from SBA-15 particles and from fibres containing the loaded particles. Only 27% of the loaded THY was released from the particles in the first 24 h and the release continued for 31 days, reaching a value around 69%. Previous reports have described different drug release kinetics from mesoporous matrices depending on the nature of the encapsulated drug. In this regard, 70–95% of the hydrophobic drug Dasatinib, loaded in SBA-15 was released in just 2 h, independent of the porosity of the materials [44]. A controlled release was obtained by Qing-Zhou et al. [45] with Ramipril, also a hydrophobic drug, having about 50% of the drug released after 5 h. Shahriarinour et al. [46] found a THY release of about 6.8% during the first hour and the release increased to 95.1% after 4 days. The sustained release achieved in our loaded materials could be related to the amphipathic behaviour of THY resulting from the analysis of its chemical structure [47]. This kind of compound, due to the hydrophobic effect, can easily self-assemble spontaneously in an aqueous media [48], forming micellar systems around silica particles, which would remain in the release medium due to the micellar concentration achievable [48]. This result was very promising; nevertheless, when loaded in PCL fibres, the release profile dramatically changed and more than 50% of the loading was released in the first 7 h. The fibres’ production process would be responsible for the change in behaviour; the DMF:DCM solvent would extract part of the loaded THY carrying it to the fibres surface and causing the fast release observed. However, the comparison with previous results for THY loaded PCL fibers (PCL-THY) [31] indicates an interesting improvement since a 55% of THY was released instead of less than 8%. Similar results were found for ciprofloxacin-loaded PCL fibers, where 30% of the drug was released in the first 10 h [49]. Due to the experimental protocol followed, i.e., replenishing with fresh solvent after sampling, a potential drug saturation in the solutions was ruled out. It is well known that complete release from electrospun PCL fibers cannot be achieved when the polymer degradation is not significant [50]. However, Srikar et al. [51] sustained that the primary release mechanism in this case would be related to desorption from the mesoporous surface. 

Taking into account the lower load in PCL@SBA-THY compared to the one present in our PCL-THY mats previously reported, the essential oil compound released in the first 24 h reached a value of around 0.03 mg of THY per mg of mat, more than twice the value found for THY loaded in PCL fibres (0.013 mg THY/mg).

### 3.3. Bactericidal Activity

The bactericidal effect of THY-loaded SBA-15 particles against *S. aureus* is shown in Table 2. THY released concentration after 24 h necessary to reach MIC and MBC were 0.07 and 0.11 mg/mL, respectively. These values were much lower than the ones previously found for free THY, obtaining MIC and MBC values of 0.2 and 0.3 mg/mL, respectively [30]. The increase of the antibacterial activity associated with encapsulation was previously reported for essential oil components loaded into montmorillonite [52]. The enhanced antibacterial activity was attributed to the slower evaporation rates of the encapsulated compounds. The encapsulation of EO compounds increases their chemical stability and solubility and the controlled and sustained release enhance the bioavailability and efficacy against pathogens [53].

The incorporation of THY loaded particles in PCL fibres leads to an increase of the released active compound concentration to easily achieve MIC and MBC values. It is well known the bacterial preference to grow on a surface rather than in the aqueous phase, and we have previously shown the importance of the direct contact between the pathogen and the THY carrier to exert antibacterial action [31]. The high bacterial adhesion of *S. aureus* to SBA-15 is favoured by the well-defined macropores size distribution [53]; however, fibrous mats, having roughness in the nanometer scale, might also promote bacteria adhesion. The difference in antibacterial behaviour of particles and fibres could be deduced by comparing Figure 1C and Figure 3B All bacteria attached to particles would be in direct contact with thymol, while just few bacteria attached to fibres would be in direct contact with THY having as one of its mechanisms of action the disruption of the peptidoglycan top layer and plasma membrane. 

However, an improvement was achieved loading the EO component into SBA-15 particles, instead of adding the free THY to the fibres preparation solution. Ten and thirty mg/mL of PCL/THY mats was necessary to reach MIC and MBC, respectively, and only 7.5 and 10 mg/mL of PCL@THY/SBA to accomplish the same results because of the larger amount of THY released, as mentioned before.

We have also confirmed the importance of the direct contact between fibres and bacteria in this case, challenging separately the exudates and the mats producing these exudates (Figure SI). As THY and CRV loaded PCL fibers, PCL@THY/SBA mats favour bacterial adhesion, locating them in close contact with the highest concentration of THY.

## 4. Conclusions

Synthesized SBA-15 rods with mean diameter of 138 ± 30 nm and mean length of 563 ± 100 nm were loaded with 70.79 ± 5.21 wt.% of THY. The essential oil compound was located inside the silica porous structure according to XRD and nitrogen adsorption results but mostly on the interparticle spaces. THY-loaded SBA-15 particles were incorporated into PCL fibres during the electrospinning process and the produced mats contained 5.87 ± 0.91 wt.% of THY. The fibres’ production process caused part of the THY present in the particles to evaporate, reducing the final THY load in the hybrid nanocomposite. Essential oil compound release from the particles was controlled and sustained for 31 days, when around 70% of the loaded THY was released. On the other hand, loaded fibres showed a burst release in the first 7 h. Again, the electrospinning process would be responsible for the dissolution and migration of part of the THY to the fibres’ surface. However, PCL fibres containing THY-loaded SBA-15 particles could double the amount of released THY from PCL fibres loaded with free THY in 24 h. As a consequence, the bactericidal action against *S. aureus* was increased, reducing the mass of mat necessary to reach MBC from 30 to 10 mg/mL. Loading THY on SBA-15 particles improves the bactericidal efficiency of electrospun mats, producing a promising wound dressing material.

## Figures and Tables

**Table 1:** SBA-15 nitrogen adsorption and THY loading results.

Sample	Nitrogen Adsorption			THY Load	Loading Efficiency ^1^
	Surface Area (m^2^/g)	Pore Volume (cm^3^/g)	Pore Diameter (nm)	(wt.%)	(%)
SBA-15	661.7	1.50	10.5	-	-
THY/SBA-15	7.3	0.05	9	70.70 ± 5.21	80.3
PCL@THY/SBA-15	-	-	-	5.59 ± 1.24	59.3

^1^ Calculated from TGA results.

**Table 2:** Antimicrobial activity of prepared materials.

Sample	MIC		MBC	
	Mass (mg/mL)	THY Released after 24 h (mg/mL)	Mass (mg/mL)	THY Released after 24 h (mg/mL)
THY/SBA-15	0.35	0.07	0.55	0.11
PCL@THY/SBA-15	7.50	0.22	10.00	0.30

## Supplementary Materials

The following are available online at https://www.mdpi.com/2079-4991/10/4/616/s1, Figure S1: Bactericidal effect of (**A**) mats after THY release and (**B**) exudates.
