# Supplementary material for: Antibacterial Effect of Thymol Loaded SBA-15 Nanorods Incorporated in PCL Electrospun Fibers
Source: Nanomaterials (Basel). 2020 Mar 27;10(4):616. doi: 10.3390/nano10040616 (PMC7221837; doi:10.3390/nano10040616)
Supplement: Supplementary file 1 [file nanomaterials-10-00616-s001.docx]

Antibacterial Effect of Thymol Loaded SBA-15 Nanorods Incorporated in PCL Electrospun Fibers

Enrique Gámez ^1,†^, Hellen Elizondo-Castillo ^1,^^†^, Jorge Tascon ^1^, Sara García-Salinas ^1,2^, Nuria Navascues ^1^, Gracia Mendoza ^2,3^, Manuel Arruebo ^1,2,3^ and Silvia Irusta ^1,2,3,^*

^1^ Department of Chemical Engineering. Aragon Institute of Nanoscience (INA), University of Zaragoza, Campus Río Ebro-Edificio I+D, C/Poeta Mariano Esquillor S/N, 50018 Zaragoza, Spain; [391519@unizar.es](mailto:391519@unizar.es) (E.G.); [gmmenc@unizar.es](mailto:gmmenc@unizar.es) (H.E.C.); helizondo_02@hotmail.com (J.T.); [626479@unizar.es](mailto:626479@unizar.es) (S.G.S.); [nurian@unizar.es](mailto:nurian@unizar.es) (N.N.); [arruebom@unizar.es](mailto:arruebom@unizar.es) (M.A.)

^2^ Networking Research Center on Bioengineering, Biomaterials and Nanomedicine, CIBER-BBN, 28029 Madrid, Spain; [gmendoza@iisaragon.es](mailto:gmendoza@iisaragon.es)

^3^ Aragon Health Research Institute (IIS Aragon), 50009 Zaragoza, Spain

^†^ These authors contributed equally to this work.

***** Correspondence: sirusta@unizar.es.


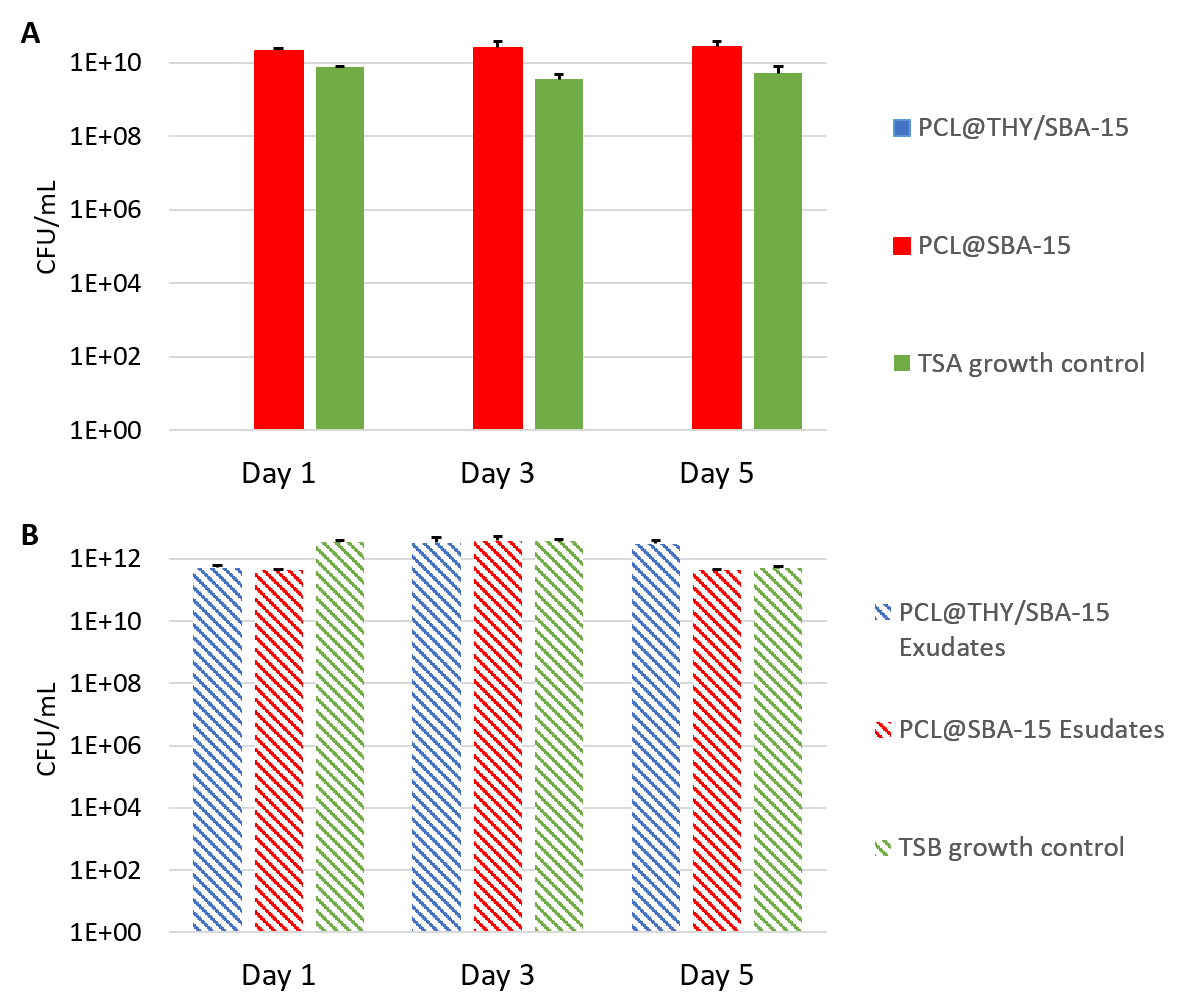


Figure S1: Bactericidal effect of A) mats after THY release and B) exudates.
